# Development and Characterization of pH-Dependent Cellulose Acetate Phthalate Nanofibers by Electrospinning Technique

**DOI:** 10.3390/nano11123202

**Published:** 2021-11-26

**Authors:** Gustavo Vidal-Romero, Virginia Rocha-Pérez, María L. Zambrano-Zaragoza, Alicia Del Real, Lizbeth Martínez-Acevedo, Moisés J. Galindo-Pérez, David Quintanar-Guerrero

**Affiliations:** 1 Laboratorio de Posgrado en Tecnología Farmacéutica, Facultad de Estudios Superiores Cuautitlán, Universidad Nacional Autónoma de México, Cuautitlán Izcalli C.P. 54745, Estado de Mexico, Mexico; dr.gvr30@gmail.com (G.V.-R.); liz_martinez@comunidad.unam.mx (L.M.-A.); 2 Departamento en Tecnología Farmacéutica, Facultad de Estudios Superiores Zaragoza, Universidad Nacional Autónoma de México, Ciudad de Mexico C.P. 09230, Mexico; dra.vicky.rocha401@gmail.com (V.R.-P.); moyz_18@hotmail.com (M.J.G.-P.); 3 Laboratorio de Procesos de Transformación y Tecnologías Emergentes de Alimentos, Facultad de Estudios Superiores Cuautitlán, Universidad Nacional Autónoma de México, Cuautitlán Izcalli C.P. 54714, Estado de Mexico, Mexico; luz.zambrano@unam.mx; 4 Departamento de Ingeniería Molecular de Materiales, Centro de Física Aplicada y Tecnología Avanzada, Universidad Nacional Autónoma de México, Campus Juriquilla, Santiago de Querétaro C.P. 76230, Querétaro, Mexico; adelreal@unam.mx

**Keywords:** periodontal disease, chlorhexidine, antimicrobial polymer, release profile, morphology

## Abstract

The aim of this work was to obtain pH-dependent nanofibers with an electrospinning technique as a novel controlled release system for the treatment of periodontal disease (PD). Cellulose acetate phthalate (CAP) was selected as a pH-sensitive and antimicrobial polymer. The NF was optimized according to polymeric dispersion variables, polymer, and drug concentration, and characterized considering morphology, diameter, entrapment efficiency (EE), process efficiency (PE), thermal properties, and release profiles. Two solvent mixtures were tested, and CHX-CAP-NF prepared with acetone/ethanol at 12% *w/v* of the polymer showed a diameter size of 934 nm, a uniform morphology with 42% of EE, and 55% of PE. Meanwhile, CHX-CAP-NF prepared with acetone/methanol at 11% *w/v* of polymer had a diameter of 257 nm, discontinuous nanofiber morphology with 32% of EE, and 40% of PE. EE and PE were dependent on the polymer concentration and the drug used in the formulation. Studies of differential scanning calorimetry (DSC) showed that the drug was dispersed in the NF matrix. The release profiles of CHX from CHX-CAP-NF followed Fickian diffusion dependent on time (t^0.43−0.45^), suggesting a diffusion–erosion process and a matrix behavior. The NF developed could be employed as a novel drug delivery system in PD.

## 1. Introduction

The electrospinning technique (ES) has recently attracted increasing interest and attention due to its functional versatility, cost-effectiveness, and potential to prepare polymeric nanofibers (NF) with applications in diverse industries (e.g., tapes, filtration technologies, energy generation, pharmaceutics, biomedical technologies, controlled release systems) [1,2,3,4].

ES has the advantage of obtaining long continuous, three-dimensional, and ultrafine fibers with diameters in the range of nanometers to a few microns (more typically 100 nm to 1 micron) and lengths up to kilometers. NF has extraordinary and unique properties such as: (a) unusually high surface area per unit mass; (b) very high porosity; (c) tunable pore size; (d) tunable surface properties; (e) layer thinness; (f) high permeability; (g) low basic weight; (h) ability to retain electrostatic charges; and (i) cost-effectiveness, among others. These techniques create new sophisticated NF types with well-defined microstructures, novel morphologies, high reactivity, and/or new functions [5]. As drug delivery systems (DDS), fiber mats have been very efficient for delivering hydrophilic and hydrophobic drugs with functional and controllable dissolution properties [6,7,8,9,10,11]. These can be used for several administration routes, including oral, topical, transdermal, and transmucosal. Moreover, NF can protect a drug from decomposition in the body before arrival at the required target [12,13]. The combination of mechanical barriers based on non-woven nano-fibrous biodegradable scaffolds and their capability for local delivery of antibiotics makes them desirable for preventing post-surgical adhesions and infections [14,15].

ES requests a high voltage electrostatic field to charge the surface of a polymer’s dispersion droplet to make NF. So that it can be described in a small series of steps where an electric field is applied, inducing electrostatic charges on the droplets of the polymer dispersion, and promoting their deformation by overcoming the surface tension, gives rise to the formation of the Taylor cone. From the tip of the cone, an electrically charged polymeric jet is ejected and elongated rapidly due to the density of the charges’ self-repulsion; simultaneously, the jet will partially or fully solidify through solvent evaporation. Finally, the jet solidifies on the collector in a random form to form nano-sized fibers [16,17,18,19].

The transformation of polymer dispersion into NF by the ES technique is affected by several parameters, including (a) the polymer dispersion properties, such as surface tension, conductivity/charge density, and viscosity, which are determined by the polymer molecular weight and polymer concentration and volatility of solvents; (b) processing condition, such as the field strength/applied voltage, flow rate, the distance between the tip and the collector, the needle tip design and placement, collector composition, geometry and take up the velocity of the collector, and (c) ambient parameters, such as temperature, humidity, and atmospheric pressure [20,21,22,23]. As long as the polymer can be ES into NF, ideal targets would be: (1) the diameter of the fibers to be consistent and controllable, (2) the fiber surface to be defect-free or defect-controllable, and (3) continuous single NF to be collectible. However, researchers so far have shown that their targets are by no means easily achievable [24,25].

NF could be synthesized from natural polymers such as chitosan, fibronectin, gelatin, collagen, silk, ethylcellulose, and others, or synthetic polymers as polylactic acid (PLA), polyglycolic acid (PGA), poly lactic-co-glycolic acid (PLGA), tyrosine-derived polycarbonates, poly ε-caprolactone (PCL), polyurethane (PU), polyvinyl pyrrolidone (PVP), polyvinyl alcohol (PVA), or their various combinations [3,26,27,28,29,30].

Cellulose ethers and esters have played an important role during the development of sustained, controlled release oral dosages as (i) coatings capable of responding to changes in the physiological environment, (ii) semi-permeable membranes that can help controlled release, and (iii) hydrophobic matrices that lower the dissolution rate of the active drug [31]. Cellulose acetate phthalate (CAP) is a physiologically inert and FDA-approved pH-sensitive polymer and has been used for decades for the enteric coating of oral formulations to prevent the premature release of the drugs in the acidic stomach while allowing drug release in the more alkaline medium as the intestine. The pH-dependence of CAP is due to the presence of ionizable phthalate groups [32]. The pKa of CAP equals 5.28 [33]; therefore, this is resistant in medium–strong acid, such as gastric fluid, and will moderately dissolve in the small intestine [34].

With the growing understanding of periodontal disease (PD), the need to develop novel technologies has resulted in the evolution of multiple advanced systemic or local/intra-pocket antimicrobial delivery systems. Nanocarriers or nanomaterials, such as liposomes, lipid, metal, polymeric nanoparticles, nanocrystals, dendrimers, and nanofibers, have been proposed as treatment options for PD [35]. However, most of the systemic antimicrobials are associated with microbial resistance with their inadvertent use, failure to reach the infection site, adequate concentration, and low tissue penetration. Due to these limitations, systemic antimicrobial delivery systems are now recommended only in severe and generalized PD patients. The use of local DDS represents an excellent alternative to treating periodontal diseases. The antimicrobial therapeutic effect is then obtained by placing the agents directly in the subgingival site/periodontal pocket. This is then released in an immediate or controlled/sustained way to combat the microbial attack, simultaneously minimizing its undesirable effects on non-oral systemic/body sites [36].

In a periodontal pocket infection, the solubility of CAP is reduced due to the acid medium and has a high solubility in a healthy periodontal pocket. It does not form a gel in the presence of water but can create pH-sensitive, controlled release, and semi-permeable systems [37,38]. Interestingly, CAP has been shown to have antimicrobial and antiviral activity [39,40]. All these properties make CAP a suitable excipient for developing new DDS that are now recognized as promising strategies for prolonging residence time and improving the specific localization of systems [41].

One of the drugs usually employed in dental clinical practice to combat and control dentobacterial plaque and its wide range of microbial species causing PD is CHX [42,43]. This is locally administered; the antibiotics administered locally allow, in turn, to reach concentrations within the periodontal pocket 10–100 times higher than those achieved with the systemic route and also with a much lower risk of causing adverse reactions or bacterial resistance in other parts of the body [44]. The main physicochemical properties of the CHX base (1,1′-hexamethylene-bis-5-(4-chlorophenyl) biguanide) are that it is a symmetric molecule with two ionizable guanidine moieties in the form of a solid white crystal (m.p. 132 °C, Mw 505.5). The water solubility of the CHX base at 20 °C is around 0.008% (*w*/*v*). Its pKa values are 2.2 and 10.3, which render it di-cationic over the entire range of physiological pH values [45,46,47]. Several groups have investigated the potential use of ES mats for the local delivery of antibiotics, such as doxycycline, metronidazole, tetracycline, minocycline, and CHX [48,49].

Different dosage forms, such oral rinses, acrylic strips, fibers, films, injectable systems gels, intra-pocket strips, vesicular systems, and micro and nanoparticle systems for periodontal applications, have been developed [50,51,52,53]. Commercial oral rinses, which contain around 0.2% CHX, are moderately effective in treating PD since they need to be applied constantly [47], they cannot reach deep subgingival tissues, even when used for an extended period, which causes treatment to be abandoned. Furthermore, the high doses required to achieve a therapeutic effect on the periodontal pocket could trigger unpleasant or even toxic adverse effects [54,55].

In this context, the development of pH-dependent CAP-NF loaded with CHX is an interesting option for the local treatment of PD. Therefore, the aim of this work was to develop, optimize, and characterize the conditions of the ES process to obtain pH-dependent NF from polymer dispersion of CAP loaded with CHX, as novel delivery systems that maintain drug concentration for long periods, decreasing the dose of the drug needed to achieve a therapeutic effect and possibly reduce treatment time, leading to greater patient acceptance in the treatment of periodontal disease.

## 2. Materials and Methods

### 2.1. Optimization of the Process of Electrospinning

The properties of the polymeric dispersion and ES process conditions employed were determined as indicated in the following sections in order to assess the influence of the concentration of polymer and amount of drug on the ES process and NF size and morphology.

### 2.2. Viscosity and Conductivity of Polymer Dispersion

Viscosities of polymeric dispersion at different concentrations (5–15% *w*/*w*) and drug (2–20 mg) were determined using a Brookfield DV-II-PRO Rheometer (AMETEK, Brookfield, Middleboro, MA, USA) at 25 °C and 5 rpm. In addition, the conductivity of polymeric dispersion and drug (15 mL) at different concentrations were measured using an Eco Test TDS Low electric conductivity meter. All results were reported as an average of three determinations.

### 2.3. The Surface Tension of the Polymer Dispersion

Surface tension was determined using a DüNoy Tensiometer modified with a Wilhelmy paddle (2.5 × 1 cm) submerged in 10 mL of polymeric dispersion at different polymer drug concentrations. The force needed to separate the paddle from the surface of the polymer solution was registered at 25 °C and it was correlated with the surface tension. All results were reported as an average of six determinations.

### 2.4. ES with Different Polymer Dispersion

ES was carried out using polymeric dispersion of CAP at concentrations among 5–12 % *w/v* in different solvent mixtures; (i) acetone/ethanol (1:1 *v*/*v*), (ii) acetone/methanol (1:1 *v*/*v*), or (iii) acetone/methanol (0.5:1.5 *v*/*v*), and in some batches, different amounts of drug (2–25 mg) were also added. CHX, which is freely soluble in the mixtures of dissolvent employed, was added to the dissolvent, following the addition of polymer, and stirred until a clear polymeric dispersion was obtained. The ES equipment consists of a high-voltage power supply, an infusion pump equipped with a syringe with a steel needle, and a grounded collector. The resulting clear polymeric dispersions were transferred to a syringe of 10 mL, with a right-angle shaped needle of 6 mm and 31 G in inner diameter attached to it. The polymer dispersion flow rate was 0.6 mL/h, applying a positive voltage range of 10–15 kV. The resulting NFs were collected on a grounded aluminum plate. The distance between the needle tip and the grounded target was 15 cm. Finally, the NFs were exposed for 24 h at room temperature to allow drying and evaporation of the residual solvent and were stored at room temperature.

### 2.5. Quantitative Determination of CHX by HPLC

The determination of CHX base was by reversed-phase adsorption chromatography with a LiChrospher^®^ 100 RP-18, 5 μm, (125 × 4 mm) HPLC cartridge (Merck, Darmstadt, Germany) on a Varian ProStar HPLC system (Chromatography Systems, San Diego, CA, USA). This device was equipped with one pump (model PS 210). In addition, the PC interface had connected an autosampler (model PS 400) and multi-wavelength UV-Visible detector (model PS 320).

Acetonitrile and 30 mM sodium acetate buffer in the mobile phase (35:65 *v*/*v*) were used to generate isocratic conditions to elution. First, the pH was adjusted with acetic acid (96%) in 0.5% of triethylamine. Then, 20 µL of the sample was injected, and the detection was made at 260 nm and a flow rate of 1 mL/min. A calibration curve for CHX was established in a range of 10–80 μg/mL. The retention time for CHX was approximately 3.5 min. Chromatograms were analyzed using a Galaxy Software package following a method adapted from Lboutounne et al. [47].

### 2.6. Entrapment Efficiency and Process Efficiency

According to the best formulations and process conditions, NFs were prepared with different amounts of CHX. Then, the amount of CHX in CHX-CAP-NF was determined by the HPLC method previously described. Briefly, a sum equivalent to 10 mg of CHX in CHX-CAP-NF was dissolved in 5 mL of MEC, then 4 mL of NaOH [0.1 M] solution was added to achieve total dissolution of polymer and release the CHX entrapped.

The aqueous and organics phases were separated, then the organic phase was dried at room temperature, and the solid obtained was resuspended in methanol to a total volume of 10 mL. A sample of this was filtered in a Millipore^®^ 0.22 μm, and HPLC analyzed the quantity of CHX in NF. Finally, the EE and PE were calculated by Equations (1) and (2).

(1) $$\mathrm{EE}\;\%=\frac{\mathrm{Amount}\;\mathrm{of}\;\mathrm{drug}-\mathrm{loaded}}{\mathrm{An}\;\mathrm{initial}\;\mathrm{amount}\;\mathrm{of}\;\mathrm{drug}}\times 100$$

(2) $$\mathrm{PE}\%=\frac{\mathrm{Total}\;\mathrm{amount}\;\mathrm{of}\;\mathrm{NF}\;\mathrm{obtained}\;}{\mathrm{Initial}\;\mathrm{amount}\;\mathrm{of}\;\mathrm{material}\;\mathrm{in}\;\mathrm{the}\;\mathrm{formulation}}\times 100$$

### 2.7. Scanning Electron Microscopy (SEM) Analysis

SEM was used to examine the surface morphology of the CAP-NF and CHX-CAP-NF. The NFs were collected on a glass surface and dried; the sample was mounted on stubs and shadowed in a cathodic evaporator with a gold layer (≈20 nm) using a JFG-1100 Sputter Coater (JEOL, Tokyo, Japan). The samples were observed under a scanning electron microscope (LV-SEM JSM-5600, JEOL, Tokyo, Japan) at 15–25 kV electron acceleration voltage and pressure of 12–20 Pa specimen chamber. The mean diameter of each prepared sample was analyzed by image analysis. The diameter of at least 30 fibers was measured for each batch, and the average diameter was expressed as mean +/− SD. Statistical analyses on diameters were conducted using Student’s test. A value of *p* < 0.05 was statistically significant and *p* < 0.01 highly significant.

### 2.8. Differential Scanning Calorimetric (DSC) Studies

Thermal properties of the NF components, CAP-NF and CHX-CAP-NF with different amounts of the drug, were assessed by performing DSC. The samples were weighed (~5 mg) directly in non-hermetic aluminum pans and scanned in a temperature range of 0–300 °C at a heating rate of 5 °C/min under a nitrogen flux of 50 mL/min utilizing a previously calibrated and adjusted calorimeter (DSC Q10, TA Instruments, New Castle, DE, USA).

### 2.9. In Vitro Drug Release Studies

The method used was adapted from that described by Silvana Gjoseva et al. [56] keeping *sink* conditions. Briefly, an amount equivalent to 0.5 mg of CHX, loaded in CHX-CAP-NF, was accurately weighed. The samples were then placed in 10 mL of phosphate buffer solution pH 7 containing Brij^®^ 58, 2.5% (*w*/*v*), and rated in closed test tubes at 37 °C using a shaking water bath at 30 rpm. At predetermined time intervals, 2 mL of medium samples were withdrawn and replaced with fresh media to complete the initial volume. The amount of CHX released from CHX-CAP-NF was determined using the HPLC method described above. The results were reported as an average of three determinations. The data obtained were fitted using the semiempirical Korsmeyer–Peppas model to evaluate the transport mechanism and the release type Equation (3).

(3) $$\frac{M_{t}}{M_{\infty }}=kt^{n}$$

*M_t_/M_∞_* is the drug fraction released at time *t*. *k* is a constant depending on the system’s structural and geometric characteristics and *n* is the diffusional coefficient related to the release mechanism and the release type.

### 2.10. Statistical Analysis

Statistical analysis was carried out using the STATGRAPHICS^®^ Centurion XVI software (StatPoint Technologies, Inc., Warrenton, VA, USA). An analysis of variance (ANOVA) between-means comparisons by a Student’s *t*-test at a significance level of *p* < 0.05 was used to evaluate the influence of the amount of drug on nanofibers’ size of EE and PE.

## 3. Results and Discussion

### 3.1. Optimization of the Process of Electrospinning

Different process parameters, such as polymer dispersion variables and ambient conditions, directly affect NF formation by ES. Therefore, the first part of this research consisted of finding the optimal ES process conditions to obtain fibers in the nanometer range from CAP dispersions at different concentrations. One of the primary process parameters that directly affect the formation, morphology, and fiber size was the applied DC voltage strength, evidenced using three different series of polymer dispersions: (i) acetone/ethanol (1:1 *v*/*v*); (ii) acetone/methanol (1:1 *v*/*v*); and (iii) acetone/methanol (0.5:1.5 *v*/*v*). The polymer dispersions were ES applying different voltages in the 7–15 kV range to observe the voltage required for NF formation.

Theoretically, an electrically driven jet liquid of low molecular weight will form droplets of small diameter. The construction of these droplets is due to the capillary breakup of the spinning jet by surface tension. For polymer dispersions, the pattern of the capillary breakup will change radically. Instead of breaking rapidly, the filaments between the droplets are stabilized. A stable beads-on-string structure was formed due to the coiled macromolecules that are transformed by elongation flow of the jet into oriented, entangled networks that persist as the fiber solidifies. The contraction of the jet’s radius, driven by surface tension, causes the remaining dispersion to form beads. As the dispersion’s viscosity was increased, the beads became more significant, the average distance between dots was longer, the fiber diameter larger, and the beads’ shape changed from spherical to spindle-like [57].

The results obtained showed that the ES process was not wholly possible when low voltages were applied in a range of 7 to 11 kV. Only fibers/NF or staple fibers are obtained using high concentration polymer dispersion. The voltage possibly is not sufficient to overcome the surface tension of the polymer dispersion, and a spraying process is successfully carried out. If the polymer concentration increases, the staple fibers become obtained. When voltages in a range between 12 and 15 kV were applied, the fibers obtained from CAP dispersion at low and intermediate concentrations (5–10% *w*/*v*) showed the presence of abundant beads in the structure. A higher polymer concentration (11–15% *w*/*v*) results in larger fiber diameter.

On the one hand, an increase in viscosity produces fiber more uniform and less beads to adequate voltage (13 kV). On the other hand, tape-shaped fibers were obtained when the voltage was very high, suggesting a modification in the surface–volume relationship. Any parameter exceeding the critical range will cause an ES failure. Hence, the importance of establishing an optimization of the variables was involved since no guide establishes the behavior followed by the different materials that can be electrospun. The optimal voltages to obtain uniform CAP-NF with little beads and a small diameter range from 11 to 13 kV.

The flow rate is another parameter that directly impacts the shape and morphology of ES fibers. When polymer concentration ranged between 9 and 12% *w*/*v*, appropriate continuous fibers with smaller diameters were obtained. Different flow rate conditions were tested (10, 15, 30 µL/min) to rule out this parameter’s influence on morphology fibers. A voltage of 13 kV was used. Hypothetically, when high flow rates are applied, this can cause a decrease in PE because there is a limit on the feed-rate per nozzle, and higher rates might result in dripping of the dispersion. Due to the force of gravity and a large amount of polymer dispersion in the nozzle, the Taylor cone cannot be maintained uniformly to obtain a stable jet due to the nozzles’ insufficient electric field and excess polymer dispersion, resulting in an inefficient process of electrospinning. Furthermore, high flow rates can cause inappropriate drying, provoking bead defects and flat, ribbon-like structures; due to that, the electric field is not enough to spin all the dispersion [58]. Due to this, it is recommended to use slow flows to allow the Taylor cone’s stable formation, from which the fibers will be expelled. After testing different flow rates, the results showed that it is possible to obtain continuous NF when the flow rate was maintained at 0.6 mL/h from the CAP polymeric dispersion with adequate concentration. This flow rate decreases the accumulation of polymer at the tip of the needle and avoids plugging.

Recently, Kim et al. (2021) reported the influence of the variation of the voltage and flow velocity on the diameter of NF. They prepared PVP-NF under specific conditions, testing voltages from 10 to 20 kV and flow rates from 0.2 to 1.0 mL/h and showed that the average diameter of NF tended to decrease when the fluid velocity decreased. When the voltages were 15 and 20 kV, the NF diameter changed but was not significant; however, the diameter increased significantly as the voltage decreased to 10 kV. They concluded that the effect of voltage on the formation of fibers is small when they are above a critical value, but when it is not reached, the instability resulting from the electric field usually has a significant effect [59].

The distance between the needle tip and the collector is another factor that affects the diameter and morphology of the NF significantly, proposing optimal distances in the range of 10 to 20 cm as sufficient spinning distance in the conventional method of ES. The length must allow the evaporation of dissolvent to obtain dry fibers and, at the same time, stretch enough to be collected in the form of non-woven mesh [30]. In our case, the optimal distance between the needle tip and the collector was 15 cm, which allowed the obtaining of dry fibers, probably due to the low boiling points of the dissolvent mixtures.

### 3.2. Viscosity and Conductivity of Polymer Dispersion

**Figure 1:**
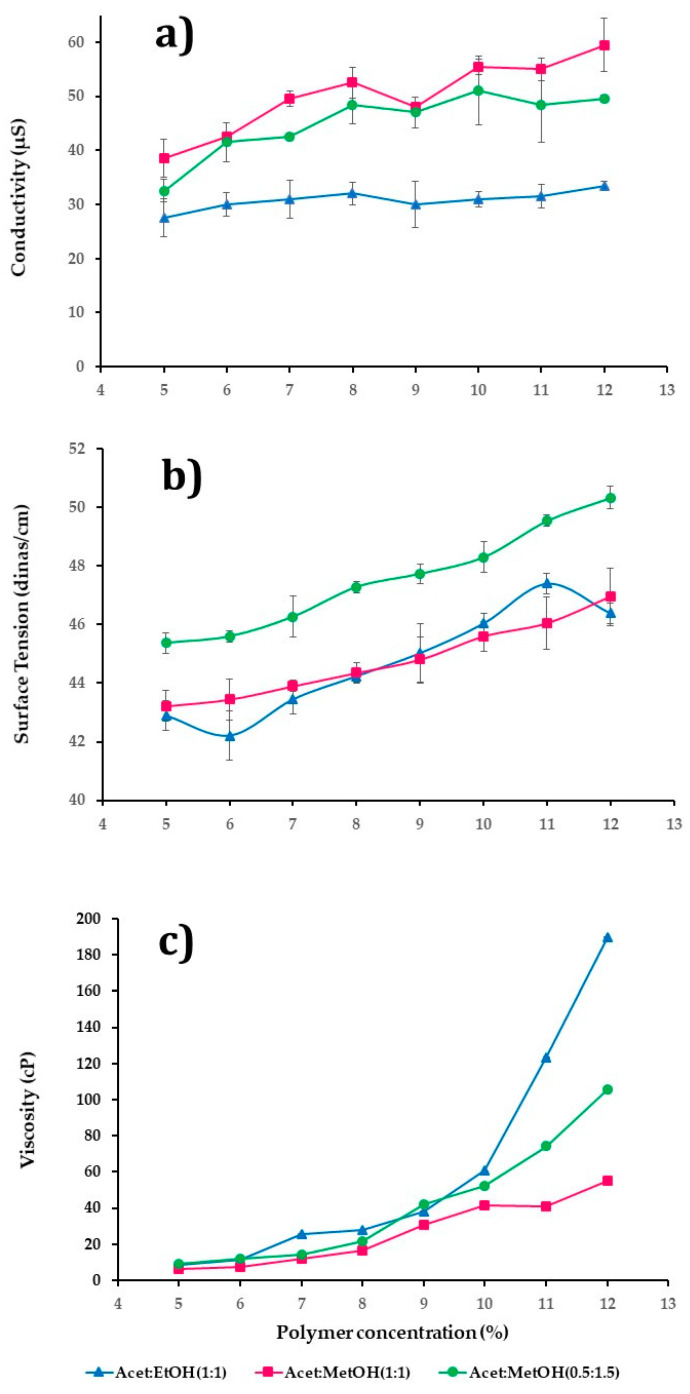
Graphs of conductivity (**a**), surface tension (**b**), and viscosity (**c**) of polymer solutions at different polymer concentrations using different solvent mixtures.

The polymer concentration affects the diameter, morphology of fibers, and PE; this aspect is too closely related to the viscosity, the conductivity, and the surface tension of the polymer dispersion. Three polymeric dispersion series were investigated to assess their influence on diameter, morphology, and fiber formation. Figure 1a–c summarized the behavior of three series of CAP polymeric dispersions in a range of 5–12% *w/v* employing different mixtures of solvents: (i) acetone/ethanol (1:1 *v*/*v*); (ii) acetone/methanol (1:1 *v*/*v*); and (iii) acetone/methanol (0.5:1.5 *v*/*v*). The conductivity and surface tension were dependent on polymers and solvents’ physicochemical properties, as Figure 1a,b shows. In Figure 1a, it was observed that there was a statistically significant difference in the conductivity with the concentration of polymer and type of dissolvent used (*p*-value = 0.0000). The polymeric dispersions that presented the best conductivity values were those whose concentration was above 10% *w*/*v*, using a mixture of solvents acetone/methanol (1:1 *v*/*v*), followed by acetone/methanol (0.5:1.5 *v*/*v*), and finally with acetone/ethanol (1:1 *v*/*v*).

Figure 1b shows a statistically significant difference in the increase of surface tension by the changes in the polymer concentration and the dissolvent type (*p*-value = 0.0000). In this case, the polymer dispersions with a higher surface tension used the mixture of solvent acetone/methanol (0.5:1.5 *v*/*v*), followed by acetone/methanol (1:1 *v*/*v*), and lastly acetone/ethanol (1:1 *v*/*v*). Thus, high surface tensions involved higher voltages in deforming the drop and overcoming this behavior even though high voltages can cause a decrease in the efficiency of the ES or the presence of beads and fibers in the form of slats.

Figure 1c shows the rheology behavior of polymeric dispersions; in general, viscosity was directly proportional to the polymer concentration in the polymer dispersion. In this case, the dispersions with the highest viscosity were those that used acetone/ethanol (1:1 *v*/*v*) followed by acetone/methanol (0.5:1.5 *v*/*v*), and lastly acetone/methanol (1:1 *v*/*v*). It is well known that the kind of solvent mixture had an effect statistically significative (*p*-value = 0.0000) on the viscosity.

The minimum concentration to produce ES NF that does not make any morphological defects is with a solvent of up to 70% dispersion mass. Therefore, only a fraction of the dispersion passing through the spinneret contributes to the NF mass-produce. The mixture acetone/methanol (0.5:1.5 *v*/*v*) has low boiling points, around 74.4 °C, that cause an increase in the viscosity of polymeric dispersion and clogging of the spinneret tip, causing a low PE.

From an optimized process as a function of the properties of the polymer dispersions, it was expected that CAP polymeric dispersions that present a better formation of NF were those with a polymer concentration between 9–12% *w*/*v*, with good conductivity properties and relatively low surface tensions. For the case of the series of polymeric dispersions that employed the mixture acetone/methanol (0.5:1.5 *v*/*v*) and above 10% *w/v* of polymer, the surface tension was high (48–50 dines/cm), which implies that a high voltage must be applied, causing the formation of fibers with beads or ribbon fibers; also, the fast speed of evaporation of the solvent mixture causes a notable reduction in EP due to clogging of the spinneret. For all the above, this formulation was discarded.

The polymeric dispersions between 9 and 12% *w/v* of concentration used the mixtures acetone/ethanol (1:1 *v*/*v*) and acetone/methanol (1:1 *v*/*v*), which were chosen for low surface tension, good conductivity, and suitable viscosity to obtain small fibers.

### 3.3. Morphology and Diameter of Nanofibers

**Figure 2:**
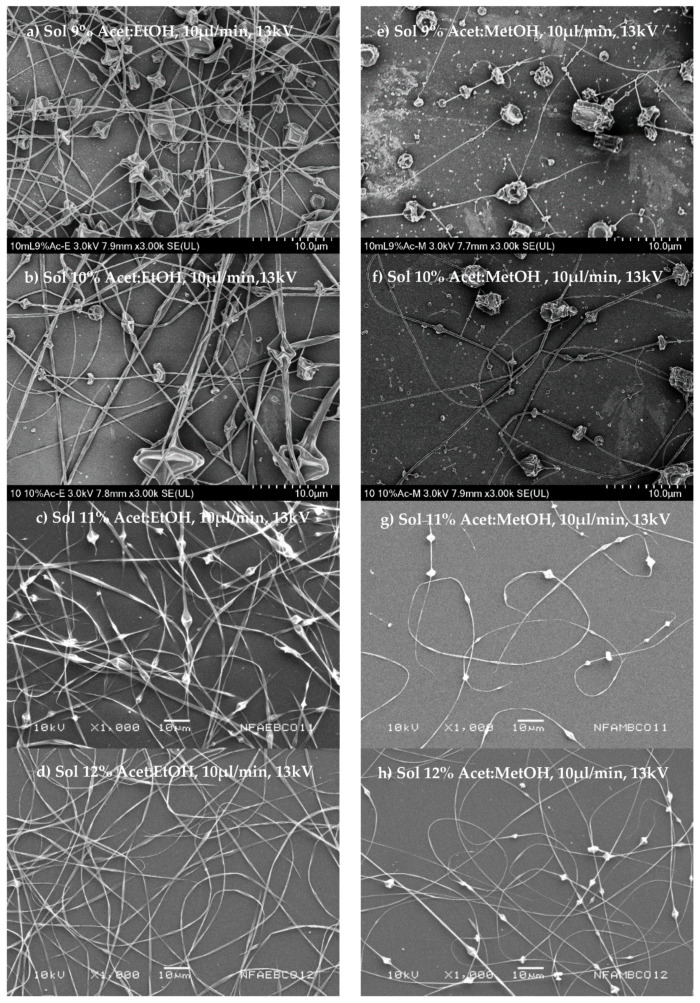
Scanning electron microscopy of nanofibers obtained from polymeric solutions at different concentrations of polymer (9–12%) and employed as dissolvent in a mixture of acetone/ethanol (1:1) (**a**–**d**) or acetone/methanol (1:1) (**e**–**h**). The conditions of the process established were a voltage of 13 kV, a flow rate of 0.6 mL/h, and a distance of 15 cm.

The morphology and diameter of the fibers obtained were corroborated using SEM. Figure 2a–h shows the morphology of fibers obtained from polymeric dispersions in a range of concentrations of 9 to 12% *w/v*, and that employed the two mixtures of dissolvents chosen, acetone/ethanol (1:1 *v*/*v*) and acetone/methanol (1:1 *v*/*v*).

Figure 2a–d shows that it is possible to obtain fibers from polymeric dispersion at low conductivity (30–33.4 µs), attributable to the relatively low surface tension (45–46 dines/cm), and an increase to dispersion viscosity in the order of 38–189 cP when the polymer concentration increases. The fibers were then obtained at the voltage necessary (13 kV) to overcome the surface tension. The high viscosity of polymer dispersion then contributed to fibers’ training due to the production of conically deformed droplets projected toward the collector. Another critical factor to consider is the type of solvent as a function of the evaporation rate at the boiling point to obtain dry and uniform fibers.

The fibers’ morphology is shown in Figure 2a–d; these show a uniform surface and a cylindrical structure for those fibers formed by polymer dispersions from acetone/ethanol (1:1 *v*/*v*) and a polymer concentration of 12% *w*/*v* (Figure 2d). On the other hand, the fibers obtained from polymeric dispersion of acetone/methanol (1:1 *v*/*v*) are shown in Figure 2e–h, demonstrating that these dispersions had higher conductivity (48–59 µs) and low surface tension (44–47 dines/cm) and viscosity (30–55 cP). Thus, the ES process was much easier with polymeric dispersions that employed acetone/methanol (1:1 *v*/*v*) as a dissolvent, implying lower energy (voltage) to induce the deformation of drop polymer dispersion from the fiber to be formed. Moreover, the dispersions have lower viscosities, greater flow, and less resistance to deformation, forming smaller diameter fibers than those obtained with more viscous dispersions. Figure 2e–h shows the fiber morphology. Figure 2e observes that at low concentrations at 9% *w/v* of the polymeric dispersion, large beads on fibers were obtained. Figure 2h shows that an increase in the polymer concentrations of 10–12% *w/v* shows that the distance between bead and bead increases, obtaining more continuous fibers. Possibly this is because the solvent mixture has a low boiling point, which causes the needle tip to drop to solidify before stretching, even when the viscosity in these dispersions is lower.

Table 1 shows the diameter means of the NF obtained from polymeric dispersions that employed acetone/ethanol (1:1 *v*/*v*) as a solvent mixture has a larger diameter (237.9–1067.2 nm) than those obtained with polymeric dispersions prepared with the solvent’s mixture of acetone/methanol (1:1 *v*/*v*), with a smaller diameter (200–466 nm). Since the type of dissolvent used was different in both cases, we can say that it influences the fibers’ diameter obtained from CAP polymer dispersions with different concentrations.

For the second part of the work and according to the previous results, only polymer dispersions whose concentration was in the range of 11 to 12% *w/v* and employing acetone/ethanol (1:1 *v*/*v*) or acetone/methanol (1:1 *v*/*v*) were used to perform this part, thus only these polymer dispersions form uniform NF. These polymeric dispersions were employed to evaluate how drug incorporation influences the properties of the solution and the ES process’s efficiency.

### 3.4. Influence of Adding CHX on NF

**Figure 3:**
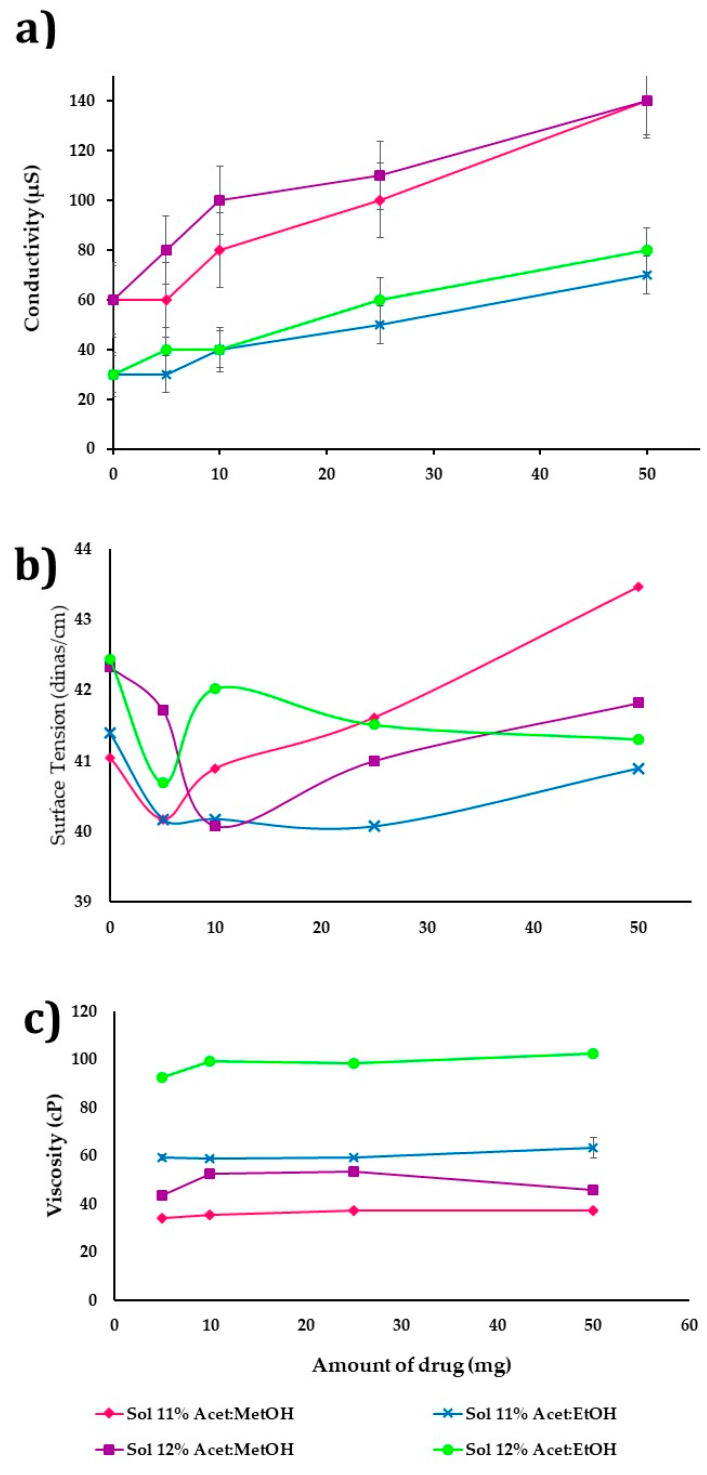
Influence of the incorporation of the drug (5–50 mg) on the properties of the polymer solutions; conductivity (**a**), surface tension (**b**), and viscosity (**c**), using polymer solutions for different concentrations and different solvent mixtures.

Figure 3a–c shows the influence of incorporating the drug (5–50 mg) on the CAP polymeric dispersions’ properties. The concentration was at 11 and 12% *w/v* and used as dissolvent acetone/ethanol (1:1 *v*/*v*) or acetone/methanol (1:1 *v*/*v*). Different amounts of drug adding and their properties concerning conductivity, surface tension, and viscosity were determined (*n* = 3). The increase in the amount of drug shows a difference that is statistically significant (*p*-value ≤ 0.05) on polymer dispersion conductivity in both cases (Figure 3a), being higher in polymer dispersion of acetone/methanol (1:1 *v*/*v*) with a range of 60 to 1400 µs, due to the CHX having a positive ionic charge, so that a greater amount of drug in polymer dispersion improves the conductivity and ES process. Moreover, the mixture acetone/methanol is more polar (conductivity 55 µs) than acetone/ethanol (conductivity 33 µs).

On the other hand, the amount of drug added does not have a significant influence (*p*-value > 0.05) on the surface tension and viscosity (Figure 3b,c). Figure 3b shows that the surface tension increases slightly (41–43 dines/cm) by drug presence. The polymer dispersions in acetone/ethanol (1:1 *v*/*v*) and the surface tension was slightly lower (41–42 dines/cm) to the greater drug amount in the formulation, while dispersions in acetone/methanol (1:1 *v*/*v*) had a surface tension of 41–42 dines/cm according to the drug increase.

Figure 3c shows the drug’s effect in polymeric dispersion on viscosity, observing that in all dispersions there is a decrease between 10 and 15% when the solvent is acetone/methanol (1:1 *v*/*v*) and 50% when acetone/ethanol (1:1 *v*/*v*) is used. If the drug amount is increased from 5 to 50 mg, the viscosity is increased slightly. Polymer dispersions at 11 and 12% *w/v* with acetone/methanol (1:1 *v*/*v*) presented values of 34 to 37 cP and 43 to 46 cP, respectively. On the other hand, the polymer dissolved in the mixture acetone/ethanol (1:1 *v*/*v*) had a higher viscosity, being in the ranges of 59 to 63 cP and 92–102 cP for concentrations of 11 and 12%, respectively (Figure 3c).

**Figure 4:**
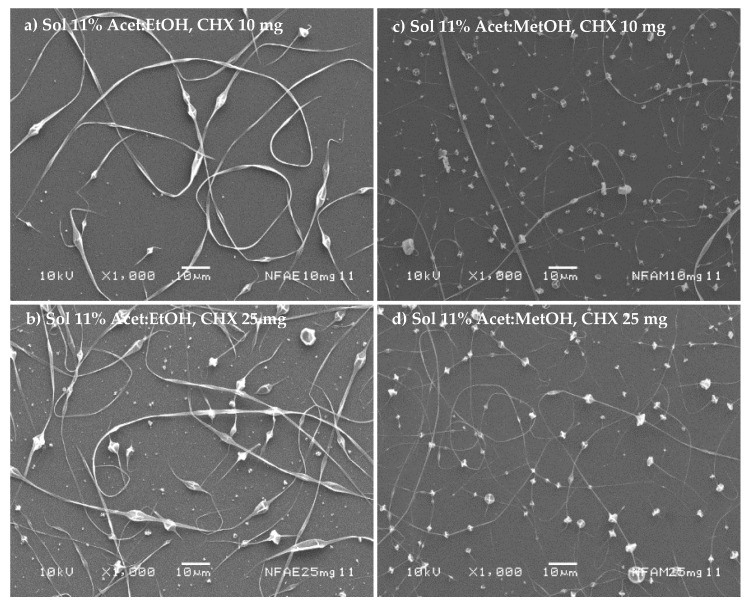
Micrographics of cellulose acetate phthalate nanofibers at 11% polymer concentration using two different mixtures of solvents (acetone/ethanol (1:1 *v*/*v*) (**a**,**b**) or acetone/methanol (1:1 *v*/*v*) (**c**,**d**)) and loaded with different amounts of the drug.

The nanofibers’ morphology is shown in Figure 4a–d considering 11% *w/v* of polymer in the mixture acetone/ethanol (1:1 *v*/*v*) or acetone/methanol (1:1 *v*/*v*) for different amounts of the drug (10 to 25 mg). In all cases, the presence of beads prevails in the structure of the fibers. Furthermore, a decrease in NF diameter was evidenced with acetone/methanol (1:1 *v*/*v*). Thus, these polymer dispersions have high surface tension values, which prevent the applied voltage from completely deforming. Furthermore, the boiling point of the solvents was relatively low.

**Figure 5:**
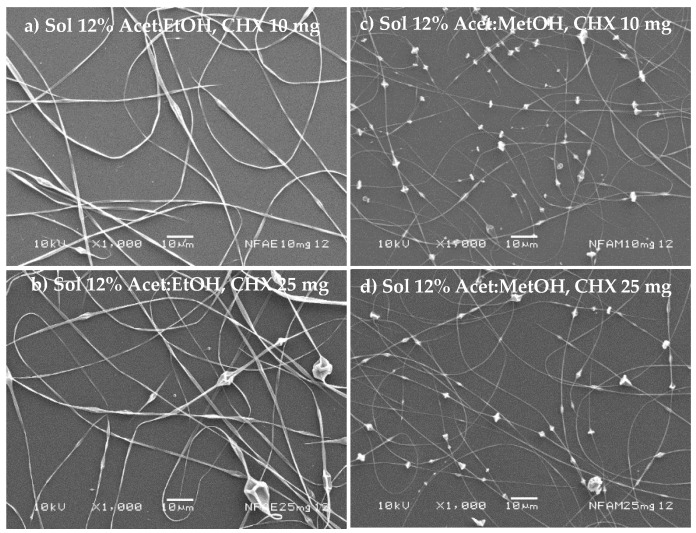
Micrographics of cellulose acetate phthalate nanofibers at 12% polymer concentration using two different mixtures of solvents (acetone/ethanol (1:1 *v*/*v*) (**a**,**b**) or acetone/methanol (1:1 *v*/*v*) (**c**,**d**)) and loaded with different amounts of the drug.

Figure 5a shows that the polymer solution at 12% *w/v* with acetone/ethanol (1:1 *v*/*v*) gave uniform NF without beads when the amount of drug was 25 mg, and NF with a low content of beads was obtained (Figure 5b). The influence of amount of drug and polymer concentration over NFs’ size was evaluated in the acetone/ethanol dissolvent system, and no statistically significant difference in the size of the NFs was observed (*p*-value = 0.8264 and *p*-value = 0.8208, for amount of drug and polymer concentration, respectively). For the case of acetone/methanol (1:1 *v*/*v*) at 12% *w*/*v*, NFs with equidistant beads were also obtained (Figure 5c,d).

Table 2 shows the mean diameter of NF obtained from polymer dispersions acetone/ethanol (1:1 *v*/*v*) or acetone/methanol (1:1 *v*/*v*) with different amounts of drug and polymer concentration. Uniform NF was obtained from acetone/ethanol 1:1 *v/v* at 12% *w/v* of polymer and drug. Their diameter was slightly larger (3 and 9%) concerning control, attributed to the dispersion property-changes when the drug is incorporated (Figure 5a). An opposite phenomenon occurs with formulations prepared with acetone/ethanol (1:1 *v*/*v*) at 11% *w*/*v*, observing a slight decrease in the diameter of NF that ranges from 2 to 12% to control. Table 2 presents the mean diameter of NF from polymeric acetone/methanol (1:1 *v*/*v*) with different concentrations and amounts of the drug, and the diameter decreased by 45% with respect to control. NF obtained from acetone/methanol (1:1 *v*/*v*) at 11% with varying amounts of the drug; the diameter was 257 nm increasing to 306 nm when 25 mg of drug was present. NF diameter increased when the mixture acetone/methanol (1:1 *v*/*v*) was 12%, increasing up to 19%. Statistical analysis showed a statistically significant difference in the diameter of NF when the amount of drug and polymer concentration varies in the formulation (*p*-value = 0.0270 and *p*-value = 0.0200) for the acetone/methanol dissolvent system.

**Figure 6:**
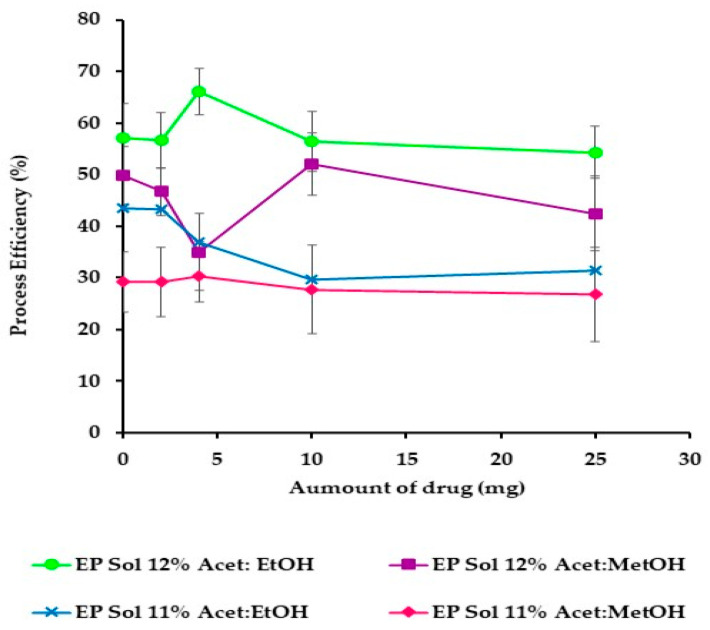
Process efficiency of different polymer solutions employed with different amounts of drug and polymer concentrations.

The ES process to obtain NF was optimized for polymer concentration and amount of drug, monitoring process, and solution-related parameters. Polymeric dispersions with acetone/ethanol (1:1 *v*/*v*) at 12% *w/v* and acetone/methanol (1:1 *v*/*v*) at 11% *w/v* were chosen to evaluate the PE and EP. Figure 6 shows that the best PE was obtained from acetone/ethanol (1:1 *v*/*v*) at 12% *w*/*v*. When acetone/methanol (1:1 *v*/*v*) at 11% *w/v* was used, low process efficiencies (40%) were obtained. Table 3 shows that PE was not affected by the increase of drug from acetone/ethanol (1:1 *v*/*v*) at 12% *w*/*v*. PE from acetone/methanol (1:1 *v*/*v*) at 11% *w/v* without the drug was 29%, but it increased until 50% when the drug was incorporated in the formulation. Thus, the increase of the drug in the polymer dispersions modified the conductivity properties, facilitating the ES process and reducing the viscosity, confirming the behavior observed in Figure 3.

The EE of CHX in NF showed decreased CHX entrapped with increased drugs incorporated in the formulation. In general, the EE decreased by up to 50% for acetone/ethanol (1:1 *v*/*v*) at 12% *w/v* of polymer and 90% for acetone/methanol (1:1 *v*/*v*) at 11% of polymer (Table 3); these tendencies are due probably to the polymeric matrix supersaturation, only part of the total amount of drug dissolving during the ES process. The rest was not entrapped in polymeric chains that would form the fiber matrix. Another hypothesis would be that droplets of polymer dispersion were exposed at the needle’s tip during the NF formation process. The voltage was generating a certain amount of heat that increases the solvent evaporation rate from the drop of polymer dispersion, decreasing their solubility capacity and thereby transporting the dissolved drug along with the fiber during the stretching process was reduced.

DSC studies were performed to understand the relations among the ingredients of the formulations. In addition, thermograms of free CHX, CAP polymer, and CHX-CAP-NF with different amounts of drug were obtained to define the physical state of the drug and the polymer in the NF and to detect drug–polymer interactions.

**Figure 7:**
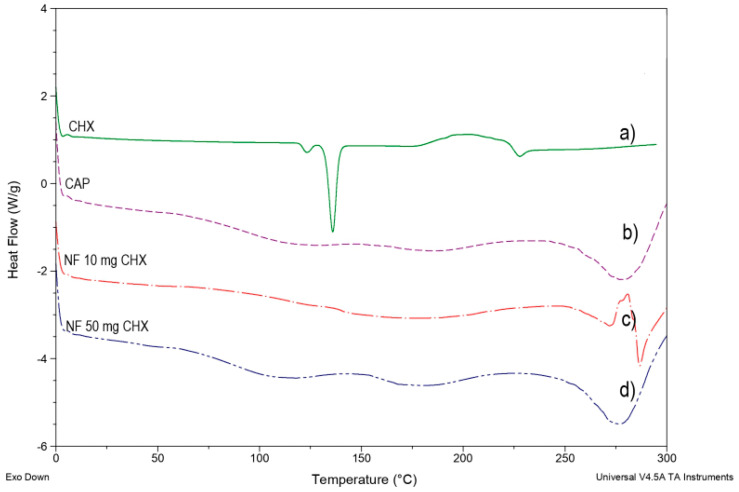
DSC of NFs loaded with different amounts of the drug. (**a**) Chlorhexidine base (CHX), (**b**) cellulose acetate phthalate powder, (**c**) membrane of NFs loaded with 10 mg of CHX, (**d**) membrane of NFs loaded with 50 mg of CHX.

The NF preparation process is simple; these consist of dissolving polymer and the drug in an appropriate mixture of dissolvent and carrier for electrospinning. However, many changes are attributable to the physicochemical drug properties before and after ES. Figure 7a shows the pure CHX melting endotherm at 135.65 °C with subsequent degradation. Figure 7b shows a slight endotherm peak around 95–100 °C followed by an endotherm peak at 175 °C due to the polymer’s crystalline part’s fusion to finally melt the endothermic peak and decomposition of the CAP polymer at 250–255 °C. Figure 7c,d shows that the endothermic peak corresponding to the melting point of CHX in any concentrations assayed was not observed. This behavior suggests that CHX was entrapped in a polymeric structure of NF composed mainly of the polymer.

Figure 4a–d and Figure 5a–d show the morphology of NF by SEM, observing that no crystal of the drug was observed on the surface of the NF. However, one significant observation was the crystalline peak of the CAP in the CHX-CAP-NF, which suggests that CHX is molecularly entrapped into the matrix of NF.

### 3.5. In Vitro Drug Release Study

**Figure 8:**
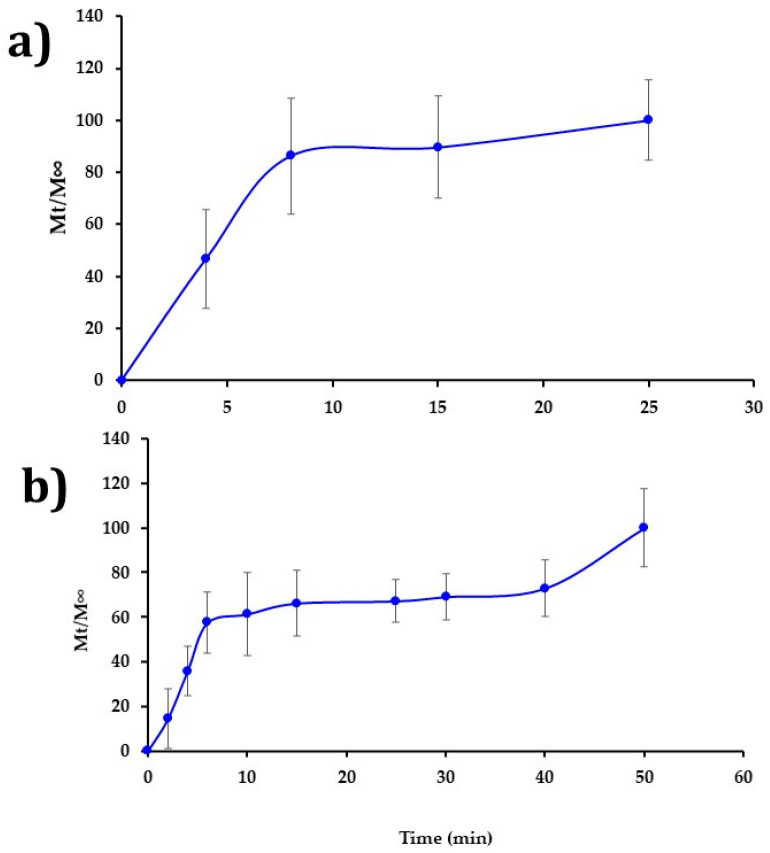
(**a**) Release profile of CHX from NFs using acetone/ethanol (1:1) (*v*/*v*) at 12% (*w*/*v*) and (**b**) release profile of CHX from NFs using acetone/methanol (1:1) (*v*/*v*) at 11% (*w*/*v*).

Figure 8a,b shows the release profiles of CHX for the CHX-CAP-NF acetone/ethanol (1:1 *v*/*v*) at 12% *w/v* (System A) and acetone/methanol (1:1 *v*/*v*) at 11% *w/v* (System B), both loaded with 4 mg of CHX. A CHX burst release was evidenced for both systems. The complete release was obtained within 25 min for System A and 50 min for System B. This difference can be attributed to the different architectures that present NF. System A has a continuous and cylindrical structure, and System B has a discontinuous cylindrical structure that presents beads, being both matrix systems. A matrix system usually releases through diffusion, and it is expected that similar behavior may occur under in vivo conditions. The Biopharmaceutical drug classification of the model drug, CHX, is into class II, low solubility–high permeability drugs, where the drug absorption is limited for its dissolution [60,61].

The results of the mathematical modeling of the in vitro release results are presented below. In both cases, System A and System B, the Korsmeyer–Peppas model presented a satisfactory fit. This semiempirical model was developed explicitly for the polymeric matrix. It is used to elucidate the release mechanism and release type because of its ability to differentiate between, and then categorize, different geometrical systems by interpreting their exponents (n). In the present case, the geometrical systems had a cylinder tendency, which theoretically means values for an n = 0.45 slab for Fickian diffusion and higher values of n, between 0.45 and 0.89 n = 0.89, for mass transfer under non-Fickian diffusion [62,63].

System A and System B showed diffusion exponents “n” equal to 0.4303 and 0.453, respectively, suggesting Fickian diffusion and time-dependent release. In order to confirm the matricial behavior, the traditional Higuchi model, used to semisolid, was applied. Good correlation was obtained for System A but not for System B. We hypothesize that this behavior may occur because System B has in its structure beads, which could dissolve after the nanofiber part stretched despite their small diameter. In some cases, the drug can act as a plasticizing agent of the polymer nanosystem that likely delays drug release in the pH 7 (Brij^®^ 58, 2.5% (*w*/*v*)) buffer solution. The medium would relax the CAPs’ polymer chains that form the nanofiber’s structure to allow drug diffusion toward the medium to a point where this becomes completely eroded. The total amount of drugs can be released.

Comparing the results obtained from both NF systems affirms that Fickian diffusion would provide the most predominant release mechanism. However, it is important to note that changes in the release medium’s pH can delay the total amount of the drug’s release time since the CAP is sensitive to changes in pH. This property could make it possible to modulate the drug release rate from the CAP-NF for a prolonged period since the pH of the crevicular fluid in the periodontal pocket changes from around 7 in normal conditions to 5 as PD progresses to inflammation and the accumulation of bacteria. As the medium’s pH (i.e., the crevicular fluid in the periodontal pocket) decreases, the CAP-NF can lower their release rate because the CAP polymer becomes less soluble at acid pH. The increase in the pH of the medium results from an improvement in the stage of PD. In various chronic periodontal diseases, the periodontal pocket’s pH that contains the crevicular fluid is slightly basic, close to 7. Still, as bacteria’s presence decreases, the disease’s stage improves until the inflammation, and other characteristic discomforts, disappear. Therefore, the decrease in pH from the bacteria on the pockets could take advantage of NFs deposited or placed directly in contact with the gums, retarding the drug release from nanosystems formulated with the CAP polymer.

Conventional pharmaceutical forms used to treat PD generally require several applications throughout the day due to the rapid elimination of the site’s drug. Indeed, only shorter intervals between administration can ensure a local pharmacological effect [64], but these additional applications come at the cost of the patient’s comfort and can affect treatment compliance. The NF proposed improving the drug’s retention and projected a high therapeutic effect in the periodontal pocket. Other areas of application, such as tissue regeneration when PD reaches severe stages, can be considered. Both System A and System B are pH-dependent. Rapid drug release is expected in chronic PD cases because the crevice fluid’s pH promotes the CAP polymer’s immediate dissolution. This occurs because the environment becomes acidic during infection due to bacterial metabolism’s combined actions and the host’s immune response [65,66].

## 4. Conclusions

The ES technique allowed the preparation of NF from polymer dispersions, where different process parameters and solution parameters influence the morphology and diameter of the nanofiber. NF systems can be obtained from CAP polymer dispersion and applied as local DDS in the oral cavity. The NF showed a better EE at a low amount of drug until 88.67% and is possibly obtained with NF of small diameters loaded with the drug. DSC studies showed that introducing CHX into the formulation decreases the glass transition temperature of the polymer. The CHX release test showed that the CHX-CAP-NF showed a burst effect, and the release mechanism was Fickian diffusion dependent on the time, which involves the diffusion–erosion process. The systems behave as material systems. Nanosystems obtained could be used in treating PD and tissue regeneration.

## Figures and Tables

**Table 1:** Diameter mean of NFs to different concentrations of polymer using two different dissolvents.

System	Acetone/Ethanol (1:1) (*v*/*v*)	Acetone/Methanol (1:1) (*v*/*v*)
Polymer Concentration (%)	Diameter Mean ± SD (nm)	Diameter Mean ± SD (nm)
9	237.87 ± 64.58 ^a,a^	200.14 ± 60.04 ^a,a^
10	413.52 ± 120.26 ^b,a^	249.01 ± 64.56 ^a,b^
11	1067.21 ± 324.46 ^c,a^	466.14 ± 181.06 ^b,b^
12	955.18 ± 188.28 ^c,a^	443.95 ± 136.56 ^b,b^

Superscript letters indicate statistically significant differences (*p* ≤ 0.05) in polymer concentration and solvents’ mixture.

**Table 2:** Diameter of nanofibers at 11 and 12% (*w*/*v*) using different dissolvents and amounts of drug.

System of Solvents and Concentration	Acetone/Ethanol (1:1) (*v*/*v*), 11% (*w*/*v*)	Acetone/Ethanol (1:1) (*v*/v), 12% (*w*/*v*)	Acetone/Methanol (1:1) (*v*/*v*), 11% (*w*/*v*)	Acetone/Methanol (1:1) (*v*/*v*), 12% (*w*/*v*)
Amount of CHX (mg)	Diameter Mean ± SD (nm)	Diameter Mean ± SD (nm)	Diameter Mean ± SD (nm)	Diameter Mean ± SD (nm)
0	1067 ± 324 ^a,a^	955 ± 188 ^a,a^	466 ±181 ^a,b^	444 ± 137 ^a,b^
10	934 ±189 ^a,a^	1049 ± 349 ^a,a^	258 ± 153 ^b,b^	324 ± 84 ^b,c^
25	1050 ± 349 ^a,a^	990 ± 99 ^a,a^	306 ± 73 ^b,b^	377 ± 84 ^b,b^

Different letters to the left represent statistically significant differences (*p* ≤ 0.05) in amount of CHX, and those on the right represent differences in type and solvent mixtures.

**Table 3:** Process efficiency and entrapment efficiency of polymeric solutions used in the elaboration of NFs.

System of Solvents and Concentration	Acetone/Ethanol (1:1) (*v*/*v*) 12%		Acetone/Methanol (1:1) (*v*/*v*) 11%	
Amount of CHX (mg)	Process Efficiency PE ± σ (%)	Entrapment Efficiency EE ± σ (%)	Process Efficiency PE ± σ (%)	Entrapment Efficiency EE ± σ (%)
0	56.94 ± 4.76 ^a,a^	-----	29.16 ± 3.87 ^a,b^	-----
2	58.21 ± 4.63 ^a,a^	72.86 ± 4.75 ^a,a^	44.90 ± 1.50 ^b,b^	88.67 ± 2.34 ^a,b^
4	55.07 ± 2.45 ^b,a^	42.60 ± 5.67 ^b,a^	40.68 ± 9.16 ^c,b^	32.78 ± 3.87 ^b,b^
10	57.19 ± 6.57 ^a,a^	35.37 ± 2.56 ^c,a^	40.49 ± 2.97 ^c,b^	29.3 ± 2.95 ^b,b^
25	56.98 ± 5.29 ^a,a^	38.00 ± 4.89 ^c,a^	43.27 ± 0.59 ^b,b^	9.28 ± 1.78 ^c.b^

Different letters to the left represent statistically significant differences (*p* ≤ 0.05) in amount of CHX as a function of a specific test, and those on the right represent differences in solvent mixtures.

## Data Availability

Not applicable.
